# Elm Bark in Surgery

**Published:** 1837

**Authors:** William A. McDowell

**Affiliations:** Fincastle, Virginia


### Art. III.—Elm Bark in Surgery.

*An account of the use of the Bark of the Slippery Elm tree (Ulmus fulva) for Bougies, Tents, Catheters and similar purposes in Surgery.* By William A. McDowell, M. D., of Fincastle, Virginia.

In 1819, the use of Slippery Elm tents was recommended to my attention by my friend, Dr. John Fleece, of Danville, Ky., as more easily introduced, more pleasantly worn, and better adapted to keep an issue or seton open, than any others. His assertions were amply sustained, by the effects produced in the case of a gentleman whom we there jointly attended.

*Lumbar Abscess.*—From a lumbar, or psoas abscess, the pus had descended to the thigh, and was there lodged, not under the fascia, as is common, but deep-seated, around the bone; of which the periosteum was destroyed. An incision, or puncture, had been made into the abscess, with a bistoury, about the middle of the thigh. This puncture was liable to obstruction, by curdly lumps, that floated in the pus, and such obstruction was always attended with great increase of tension and pain. The case seemed hopeless; but an enlargement of the fistula was obviously proper, and the patient objected to the use of the bistoury. It was in this situation, that the alternative of a Slippery Elm tent was determined upon.

A strip of the seasoned inner bark, long enough to reach the bone, and to leave an external portion to be bent down over the skin, (by which it could be withdrawn) smoothly polished, rounded at the point, and shaped as nearly to the size of the puncture as we could get it, was dipped for a few seconds in tepid water. Covered with mucilage, and so slippery that it could with difficulty be held in the fingers, it was introduced into the sinus; where it remained 12 hours, without (according to the patient's report) producing one disagreeable sensation. On its removal, the sinus was found to be enlarged from one-fourth to one-third of an inch, by the expansion the seasoned tent had undergone. Another tent was now prepared, of the size of that just removed in its expanded state, to be next introduced; and so on, in succession, they were used larger and larger, until the opening was made as wide as seemed necessary, without pain, or effusion of blood.

*Mammary Abscess.*—Tents of this description, I have found particularly advantageous in the treatment of mammary abscess.

In cases of deep-seated abscess, but not answering the description of a disease under that name, as given by Mr. Hey; (such I never saw); but in cases, or more especially in a case, of long standing, with deep sinus, dipping into and through the mamma; with the gland in an indurated, rough, and enlarged condition; I was completely successful, by tenting the different sinuses.

A case of this description had been pronounced cancerous, and I was employed on account of the belief that extirpation must be performed. The tents were made thin, and softened in water, until their flexibility admitted of their following the devious course of the sinus, without the application of force or violence. In six or seven days, the secretion of healthy pus commenced throughout the sinus.

The external openings were enlarged in this, and in most cases, through the skin, with the point of a lancet, previous to introducing tents. After the secretion of healthy pus occurs, the tents should still be made as wide as ever, but a little shorter every day or two, and thinner at the point, and at the sides, that no violence may be done to the granulatious.

*Gun-shot Wounds.*—In all abscesses, sinuses, or wounds, that require tenting, the Slippery Elm merits a preference. To widen a sinus, or to prepare it for the extraction of loose bones during the process of exploration, their superiority is obvious.

A young man of this town, walking behind a companion whilst hunting, and almost in contact with the muzzle of his gun, received the contents of his musket, shot and wadding, (accidentally discharged) in his shoulder. About an inch of the centre of the clavicle was shattered, and the fragments carried deep into the wound. Some of the shot passed out through the scapula, and some posterior to and below the middle of its basis; the rest, together with most of the wadding (which was of dry leaves,) and portions of the clavicle, lodged against its flat side. This wound was tented, with broad and thin tents, to keep the opening wide, yet not to prevent discharge, until all the foreign matters were removed with slender forceps, and until the wound healed from the bottom outward.

*Strictures*—*Case* 1*st*.—Jan. 13, 1831, I visited Mr. C.—— at his father's, in this country, near Haweytown. I found him shut up in a stove room, heated to about 100 degrees, and rolled in blankets, yet tormented with rigors; his emaciation extreme, complexion chlorotic, tongue furred, and countenance unhappy. Mr. C—— (now Dr. C——, of Indiana,) had some weeks previously returned from North Carolina, where he had been advantageously engaged in gold-mining, until he was interrupted by the violence of the disease. It had been gradually increasing for more than three years; within which time, he had been under the management of several physicians—some of them eminent. They afforded him none, or but very transient relief. From his mines he visited Charleston, and several intermediate places, in quest of surgical aid; but deriving no benefit, he returned to the mines in despair, sold them, and relinquishing all his golden expectations, came back to Bath-Court, to die among his kindred. On arriving at his father's, he put himself under the care of a physician, whom I conceive to be inferior in professional ability to but few in Virginia. After enduring his management for about four weeks, Mr. C—— abruptly dismissed him and sent for me. He said Dr. —— had cauterized him nearly to death, and in despite, too, of his repeated protestations, that this plan had been tried over and over before, and had failed; and he even shuddered as he spoke of his sufferings. The stilet, I learned, had also been used with no better effect.

I inserted an ordinary bougie, of which he had a variety, and at the distance of about three inches, met with a stricture opposing firm resistance: the largest bougie it would admit was smaller than a crow's quill. Not knowing exactly what to do for the stricture, I did nothing. For his general health, I prescribed a purgative of salts and magnesia, and a blue pill of five grains, to be taken daily; a hip-bath occasionally; and a gradual reduction of the temperature of his chamber; leaving him with a promise to visit him again in a few days.

In the course of my reflections on the case, it occurred to me, that one of those expanding Slippery Elm tents, might be shaped into a bougie, that would expand and cure this stricture; and I forthwith rode out and made the trial. A piece of dried inner bark, shaped to the size of a bougie that would pass the stricture, and slightly tapered, was, after remaining a few seconds in tepid water, passed through the stricture; pushed firmly into it; and suffered to remain four hours. It produced less uneasiness than was anticipated: of pain there was none, and the uneasiness seemed to be overbalanced by the mental relief arising from the contemplation of a new project. On removing the bougie, which required a severe pull, a perfect impression of a stricture, from a fourth to a third of an inch broad, was indented around it; rough, and pitted on one side, as if it had been impressed with a thimble. The bougie had expanded considerably, except that portion of it which was embraced by the stricture. There it appeared but little enlarged, and the bulge beyond had caused the difficulty of extraction. I now prescribed the introduction of such a bougie, every day or two, to be retained as long as it could be tolerated; a blue pill to be taken every third day, and the bowels to be kept open with saline purgatives. The dilatation was at first very gradual; but in ten or twelve days, so large a bougie was required, that it became necessary to double the bark, by gluing the flat sides of two pieces together. On the 17th February he was dismissed cured, and has never since been troubled with the disease.

The slow progress (when compared with any other case that I have since encountered) and difficulty, in the dilatation of this stricture, I have ascribed to the oft-repeated incisions with the stilet, and the ulcerations from the caustic, having converted its inner surface into a cicatrix, with diminished expansibility.

The bougie here recommended, and brought by this case directly in comparison with the caustic and the stilet, it must be evident to any one acquainted with these diseases, is not calculated to supersede either of them; and I recommend it only as an additional agent in treating strictures; yet, in a great majority of cases, it is decidedly superior to either, or to any other plan of which I have any knowledge. It is particularly adapted to all strictures, proceeding from spasmodic contraction, or from circula thickening of the membrane, or from callosity, or indurated enlargement of the surrounding corpus spongiosum, or of the prostate gland.

But in case of obstruction from a thickening of one side of the urethra, projecting as an excrescence into the canal, in the form of a wart, I can conceive nothing short of extirpation to be adequate to effect a cure; and no plan strikes me, as so eligible for effecting this, as the application of caustic. The stilet would be wholly inadequate, and from the expansive bougie, only temporary relief could be anticipated. It is true, that tumors, even warts, may sometimes be removed from the surface of the body by pressure; but it must be long continued and firm; to a degree not to be endured on a structure so delicate as that of the urethra. In cases of total obstruction, from filling up, or obliteration of a considerable length of the urethra, the stilet is, I conceive, without a rival; and we have no alternative to it but incision trough the integuments.

*Case 2d.*—July 9th, 1828, I visited, in the night, Isaac, aged about 55 or 60, a negro man, belonging to Gen. Carington, of Halifax county, who was at the General's plantation, in this neighborhood. I found him suffering much from retention of urine; the bladder much distended, pulse full and hard, and his tongue furred. He had passed no urine, except in dribs, and in all amounting to but very little, for more than 48 hours. I bled him copiously, and immersed him in a hip-bath. No relief being obtained, I attempted the introduction of a cathetic; but was not able to accomplish it. On introducing my finger in the anus, to direct the instrument, I found it had stopped at the prostate; which appeared to be of the natural size and form. I withdrew the instrument, and inquired if he had been subject to such attacks. He said he had, for about eight years; not so frequently in former times; but that they increased on him, so that he lately had an attack every two or three months, though they were not often so violent as in the present case; that some of the physicians in Halifax had frequently attempted to introduce the instrument I had just tried, but none could succeed; that he had not been able to labor for three or four years, and that his urine, at all times, dribbled away from him. I repeated the bleeding, carrying it nearly to syncope; administered emollient enemas; repeated the hip-bath several times, and exhibited full and frequent doses of sp. nit. dulc., which, in the course of the night, relieved him. I then prescribed saline purgatives daily, the hip-bath and nitre occasionally, with a decoction of mallows for constant drink, until he got over the attack.

I learned from the overseer, that Isaac had been a few weeks since sent by his master from Halifax to this plantation, to be maintained pretty much as a pensioner on the estate. From this time, until June 1831, I frequently attended him in such attacks, and in their intervals made several trials with the bougie, on the plan recommended by Mr. Abernethy. They did no good, and my patient informed me, that in this, too, I had been anticipated by physicians in Halifax.

June 2d, 1831, I visited Isaac in one of his usual attacks, the first since my experiment on Mr. C——. After a few days spent in relieving pain, and allaying inflammatory action, I introduced a Slippery Elm bougie into the bladder. It produced very little irritation. He retained it more than five hours, and sat, stood, or walked about most of the time. On withdrawing it, blood flowed for a few minutes, and an impression of a stricture about one fourth of an inch broad was made on the instrument. I shaped another bougie to the size this had attained by expansion, and directed him to come to Fincastle as soon as soreness from this operation subsided, that I might repeat it. No soreness followed; and with four bougies, and within a week, I succeeded in dilating this stricture somewhat beyond the natural calibre of his urethra. It required a moderate pull, on withdrawing the last bougie, through the whole length of the urethra, from the prostate gland to the orifice. There has been no return of stricture; he has enjoyed, ordinarily, good health, and makes as good an operative as any man of his age on the plantation.

Since 1831, I have used no bougies but those of Elm bark. I

examine strictures with steel sounds of various sizes, and have invariably cured them with elm bougies. But not always with the facility and despatch indicated by the foregoing cases. Several cases, recent and of less historical importance, have given me much trouble and perplexity. As in the following example:

*Case 3d.*—Mr. —— consulted me March 30th, 1834. He had labored under gonorrhœa since June 1832, and in August 1833, suffered long and severely from swelled testicle; since which time his gonorrhœa had frequently ceased, sometimes a week or two, and recurred again. For a while previous to his application to me, he had discovered, that the stream of urine was lessening, and that it was occasionally voided with great difficulty, dribbling away, and now and then totally obstructed for several hours together, causing him much uneasiness. His gonorrhœa was purulent; but whether he might not have renewed it occasionally in the time, he could not satisfactorily decide.

After he had urinated and used the syringe, I proceeded to examine the urethra. At about 2 1-2 inches was a stricture, admitting a very small bougie. One made of elm bark, a little tapered, was introduced. It produced uneasy sensations, and a shuddering; but in 10 or 15 minutes he was sensible of no uncommon feeling; and on examining the bougie, I was able to push it several inches farther in. It was soon loose again, and was withdrawn, and a larger one substituted. This remained three or four hours, and when withdrawn, exhibiting a thread-like mark of a stricture. Next day another was introduced, which expanded it to the full natural size. On attempting now to pass a sound towards the bladder, I encountered another stricture, at the junction of the membranous portion of the urethra with the bulb; which I concluded to dilate next day; but on examining then, I found it was gone; the urine flowed in a full stream, all uneasiness had ceased, the gonorrhœa had almost stopped, and in a day or two was well. He remained apparently well two weeks or more, and then began occasionally to feel uneasy sensations when urinating; and, finally, the difficulty of voiding, and smallness of the stream, caused him to consult me again. April 18th, I found the stricture nearly as narrow as before, but quite as easily dilated; and the cure seemed again as perfect. The same occurred afterwards, May 10th, June

3d, and finally July 5th. At this time I shaped my bougies larger and larger each day; giving a bulge to the part to be fitted into the stricture, until the last bougie, when withdrawn in its expanded state, exceeded half inch in diameter; yet this gave him no more pain than others, nor any other feeling, than a sense of numbness in the part. Acute pain, however, was experienced when the expanded portion passed out of the orifice. He since remains well.

*Fistula in Perinæo—Case 1st.*—In Oct. 1824, I visited a negro boy aged 12, the property of Mr. M. of this county. He had several fistulous openings in the perinæum, through which he passed all his urine. There had been considerable sloughing of the part, and that process still continued. On passing a sound into the urethra, I found the part within the scrotum filled with a calculus. I pushed it back, enlarged one of the sinuses, and extracted it. I prescribed a course of treatment calculated to produce present relief, and requested his master to send him to town as soon as he was well enough for farther treatment. He was so much relieved, that he was not sent until the following spring; when on the 9th May, I made incisions through the callus of the fistulas, from the perinæum to the urethra, and suffered him to pass no urine without a catheter, until the part had healed. When it was found that all his urine flowed by the natural channel, I permitted him to return home; but advised that his urine should still be passed by the catheter for a few weeks; which unfortunately was neglected, and a fistulous opening made its appearance again. The disease progressed, and was unattended to, until May, 1834, when he came to town. There were now several fistulas, proceeding from different orifices in the membranous portion of the urethra. From the anterior sinus forward, the urethra was totally impervious for the space of at least two inches—filled with a grisly callus. He was much reduced, and labored under hectic, with copious night sweats.

Before proceeding to any operation, except to widen, externally, one of the fistulas, that the urine might descend with less interruption, I kept him 13 days on a course of alteratives and tonics. The time was more than was requisite; but I had difficulty to determine in what manner I should operate, or whether I would operate at all. The negro's supplications at last determined me in the affirmative; and I proceeded to make an opening through the obliterated portion of the urethra, with a stilet. A silver catheter was then introduced into the bladder, and the fistulas were incised, the wound dressed, the silver catheter removed, and one of elm bark introduced into the bladder, and then kept fixed, until all was healed. On the twenty-first of June, he returned home in good health. A small fistula has since occurred, and remains, but does not increase, nor impair his health. This man never kept his bed one entire day after the operation; but walked the streets more or less every day during his stay in town, with the catheter in his urethra; from which he never, during the time, complained of either pain or uneasiness.

The catheter of elm bark is thus prepared. I take a thin strip of the inner bark, from 1 to 1 1-2 inches wide, seasoned just so much as not to destroy its pliability, level the edges, and smear them with mucilage or glue; wrap the bark either spirally or longitudinally around a stilet, and roll with tape. The wire should be smeared thinly, but completely, with beeswax and tallow, to prevent its being retained by the glue. A good mechanic could make a nice article of this kind, which for the purpose of being left in the urethra, when a case may require such treatment, proposes great advantage over any other catheter, for when thus left, coated with mucilage, instead of acting as an irritant, it proves a fine emollient to the inflamed or lacerated parts.

*Case 2d.*—In January, 1835, Dr. W——, whilst on a visit at Buchanan, was taken with pain and swelling between the anus and ischium. He had suffered from a stricture of the urethra, at the bulb, for about two years. He had used bougies, but never had resorted to the stilet, or caustic. It had progressively grown worse, and for some time past, he had voided urine with extreme difficulty.

I visited him, January 7th, in company with Dr. Reed, of that town; examined the stricture, ascertained its calibre, and pushed an elm bougie into it: he became faint, and suffered much more than I ever witnessed from the instrument in any other case. The stricture dilated readily, but left on the bougie its impression for about one-fourth of an inch in width. His urine flowed after it with ease, and in a pretty full stream. On the following day, a full-sized bougie was inserted, and suffered to expand. It gave no pain. After withdrawing it, I attempted to introduce a metalic catheter, but could not effect it, although it evidently had passed the stricture which was at the bulb. Examination of the prerinæum, and *per anum*, satisfied me, that it had entered a sinus, and I now plainly traced the swelling and hardness, which were near the ischium, on to the urethra. On the 12th January, Dr. Reed opened an abscess near the anus, and discharged a considerable quantity of pus and fetid urine. No other incision was made into this sinus, than this simple puncture, with an abscess lancet; no catheter was worn, or even introduced into the bladder; yet no more urine passed by the sinus, and it healed kindly, and was entirely well, without farther attention, in 8 or 10 days; his urine flowing boldly.

This case has induced me to believe, that the great difficulty generally encountered in curing *fistulæ in perinæo*, both recent and chronic; but especially if recent, may, in many cases, be consequent, as much on incomplete removal of the stricture that caused it, as to callosity, and change of action in the fistulæ.

*Fistula Lachrymalis.*—I have treated three cases of this disease with slippery elm bougies. In two of them, a slender probe could be passed, from the abscess, through the *ductus ad nasum*, into which bougies were introduced. Cures were effected in each case in about a fortnight.

The other case was that of a lady, whom I attended in the spring of 1833, at James River forge. She had labored under the disease for several years. The duct was completely obliterated. I perforated the callus that filled it, with a sharp, triangular pointed probe, and inserted successive dried elm bougies, until it was dilated to the full natural size. I then kept it tented for four weeks, with bougies of the *fresh bark*, before the external opening was suffered to heal. The cure is perfect.

Several physicians in this neighborhood have, on my recommendation, treated strictures with those bougies, and express themselves as well satisfied of their superiority as I have been.

*Calculus and the Operation of Lithontritie.*—There is no aspect in which I have ever considered in a more favorable point of view, the benefit which is likely to result from the expanding property of the slippery elm bougie, than in relation to Civiale's operation of lithontritie.

I cannot but indulge the pleasing anticipation, that with such assistance, the invention of that great benefactor of his race, must eventually set aside the gorget and the staff, as a forlorn hope, to be retained only as a dernier resort, and not to be required to achieve a triumph over calculus once in an hundred cases.

The power of a lithontritor, from a half to three-fourths of an inch in diameter, upon calculus in the bladder, needs no description. It is easily conceived in idea; and yet it is no mere ideality, or idle creation of the fancy, to believe that one of that size may be introduced. The urethra can be so expanded as to admit such a lithontritor. That it can, will be evident to any one who will duly consider the anatomy of the part, and the size of calculi that have passed through it. And, moreover, I have dilated it with bougies, to an extent exceeding the minimum of those measures, with but little effort, and without producing pain.

I have now in my possession a calculus, the greatest transverse diameter of which is six-tenths of an inch, the least, or that of the flat side five-tenths, the longitudinal eight-tenths, carefully measured, that passed spontaneously from a female patient in 1825, whilst using antilithics and diureties; viz. aqua calcis, spts. nitre, and decoction of mallows. Arterial action was kept down by blood-letting. Sir A. Cooper extracted eighty through the urethra, with forceps. The urethra indeed seems constructed for expansion, almost as perfectly as the vagina; it is full of longitudinal folds or rugæ, which when simply unfolded, more than doubles its dimensions, even without drawing upon its elasticity, which is as great as that of any other part of the body; for we see it contracting into strictures, from slight irritation; and expanding into sacs, of astonishing dimensions, by the force of the urine, impelled only by the moderate contractile power of the bladder; and the division of the prostate gland into lobes and its spongy texture well adapts it for yielding to dilatation.

I think it difficult then to say, to what dimensions it would be unreasonable to suppose the adult urethra might be dilated, by the very gradual expansion of the bland emollient material here recommended.

Over half an inch would not, I can declare from experience; and it was with a view to be able to speak more experimentally, on this portion of my subject, that I so long delayed to communicate an improvement, that I consider so important in treating strictures, with the expectation, that I might encounter a case of calculus, that would justify a dilatation to the extent that the membrane was capable of bearing. But this is not the region for calculus, and I have neither met with, nor heard of a case, since commencing the use of this material for bougies.

The process of dilatation, with this substance, is calculated to prove beneficial too, not merely in affording more room for action, but by lessening the irritability and sensibility of the parts, and thereby preparing the patient to endure a lithontritor for a much longer time, as well as of greater size; in illustration of which I will here adduce a few facts. It is familiar to every one who has been under the necessity of introducing catheters or bougies, daily or oftener, that the first introduction is the most painful, and that each successive operation becomes less and less so, until at last nothing more than a pleasant titillation is experienced, but little, if any, greater in degree than was formerly experienced from the passage of the urine. And such is the case with every part of the body, as illustrated in the pituitary membrane, by the snuff-taker, in the retina, by the refiner and the glass-blower, and in the soles of the feet, by the pedestrian.

Independent of any effort at dilatation, we are informed, that Civiale always introduced a catheter or sound, for several successive days, previous to operating with the lithontritor, "to lessen irritability," and generally suffered it to remain in the urethra for several hours, and more especially, when the parts were unusually irritable. In one very irritable case, which he has related, the first introduction of the sound gave very great pain; but after operating with the lithontritor, so much had sensibility been diminished, that the passage of sand and soft gravel gave none.

A lady, upon whom I had performed the operation of lithotomy, in 1819, in Danville, Ky., had used anti-lithics, prescribed by quacks, by injection through the urethra—caustic alkali, among others—until the sensibility was so far impaired, that she was insensible to the contact of any thing that was applied to the part.

The surgical works and medical periodicals of latter years, abound in redoubtable obstacles to Civiale's operation; most of which have reference to the irritable state of the bladder and urethra, from the long continued pressure of calculi. An answer to all of which, I conceive to be comprised in my preceding remarks on irritability. Others are worthy of more particular consideration, among which, enlargement of the prostate gland is generally advanced, as constituting an insuperable obstacle. The difficulty of introducing the instrument here seems to be the insurmountable part of the process. Now this *can* be surmounted by the elm bougie; but the question recurs, would it be advisable? In answer, I will venture as a surmise, that the pressure, from long continued efforts at dilatation, might chance to excite such a change of action in the part, as to effect a cure of the gland. A trial, surely, could not be amiss in a disease which is reckoned incurable. I have witnessed such results in indurated mammary glands, and why should I not in similar affections of the prostate? The effect of pressure made by bandages on external indurated parts is a familiar illustration.

Although I can admit no urethra to be too irritable, or too tender, no plurality of calculi too numerous, nor any stone to be too large for lythotritic, with a properly dilated urethra, yet there are cases wherein the lythotomist must come in aid. Patients who have stones that are encysted, I should not conceive to be safe subjects for lithotritic. But this is rare, and a surgeon who is master of his business can always detect it with the sound, before proceeding to any operation. And calculi sometimes, though still more rarely, adhere to the bladder. Morgagni gives an account of a large stone, with part of its surface thus attached. In the case of a gentleman on whom I performed lithotomy in Kentucky, a stone, the size of a hen's egg, adhered to the bladder with such firmness, that it was extracted with great difficulty. On coming away, about one-fourth of its anter lamella was wanting, and was found attached to the bladder, so firmly that it required time and considerable force with the scoop and fingers to detach it. Such cases would be detected with a sound, or with the lithontritor, by turning the instrument once or twice round, after grasping the stone. A precaution that should, at all events, and in every case, be observed, to gain assurance that no portion of the bladder was grasped, with the stone, by the forceps.

Those two situations *alone* appear to me as exclusively requiring the operation of lithotomy. Other contra-indications that have been arrayed against lithotritie, such as diseased bladder, scirrhus prostate, severe affections of the kidneys, or of important vital organs, or calculus in very old men, with glary urine, &c., are, I conceive, equally inauspicious to either, and every operation.

Some caution is necessary in using bougies or catheters of elm. Although this bark possesses a degree of tenacity surpassed by that of but few trees of the forest, yet when seasoned, and in a very dry state, it would be liable, in the hands of a careless, or awkward operator, to break off in the urethra or bladder. To obviate this danger, it should be immersed in water, for a longer period when it is very dry, which will restore tenacity to its outer fibers.

October 1837.

